# Alliance Ruptures and Psychotherapy Outcomes: A Multilevel Meta‐Analysis of Their Association

**DOI:** 10.1002/jclp.70192

**Published:** 2026-07-17

**Authors:** A. Babl, J. M. Gómez Penedo, C. Nimphy, M. Keßel, C. Crainic, N. Boendermaker, C. F. Eubanks

**Affiliations:** ^1^ Leiden University Leiden the Netherlands; ^2^ Free University of Brussels Ixelles Belgium; ^3^ Gordon F. Derner School of Psychology Adelphi University Garden City New York USA

## Abstract

**Objective:**

Although two meta‐analyses have examined the relationship between rupture repair and treatment outcome, no meta‐analysis to date has investigated the association between alliance ruptures and treatment outcome. Numerous primary studies have explored this relationship; however, the methods used to assess ruptures vary considerably. These include direct measures, such as ratings of the occurrence, frequency, or intensity of ruptures by observers, patients, or therapists, and indirect measures, such as fluctuations in alliance ratings. This methodological heterogeneity may contribute to inconsistent findings across studies. The present meta‐analysis sought to clarify the association between alliance ruptures and treatment outcomes by including only studies that assessed ruptures using direct measures.

**Methods:**

After applying inclusion and exclusion criteria, four publications remained reporting 18 effect sizes of the associations between ruptures and outcomes. Those provided data from *N* = 301 clients undergoing psychotherapy. Multilevel models with a three‐level structure with restricted maximum likelihood to estimate the aggregated effect size of ruptures on outcome were conducted.

**Results:**

The results showed a significant aggregated effect size of *z* = −0.22, *SE* = 0.04, 95 CI [−0.303, −0.133]; *t*(17) = −5.40, *p* < 0.001, *r* = −0.21, indicating that alliance ruptures were associated with poorer therapy outcomes.

**Conclusions:**

A small but consistent negative association between alliance ruptures and treatment outcomes was found. An individual patient data meta‐analysis would be an important future endeavor in the field of alliance ruptures, by including more studies and a homogenous analytic approach to estimate rupture–outcome associations and including rupture–repair as a moderator.

1

The therapeutic alliance, defined as the collaboration on the tasks and goals of therapy as well as the affective bond between client and therapist (Bordin 1979), has been found to be predictive of treatment success (Flückiger et al. 2018). However, the alliance is not a fixed phenomenon, but is rather characterized by rupture and repair episodes, which are common across theoretical frameworks and therapists of all skill levels (Eubanks‐Carter et al. 2012). Alliance ruptures are defined as breakdowns in the collaboration between therapist and client on tasks or goals or a strain in the emotional bond (Eubanks and Muran 2022). Ruptures can be marked by *withdrawal*, *confrontation* or a combination of both (Safran and Muran 2000). In the case of withdrawal ruptures, the client and/or therapist moves away from the other, or the work of therapy, while in confrontation ruptures, the client and/or therapist moves against each other or the work of therapy (Eubanks et al. 2018). Alliance ruptures can have detrimental effects on therapy outcome or may lead to dropout (e.g., Rubel et al. 2018).

Ruptures can be addressed by means of repair strategies. Repair strategies are interventions that aim to resume collaboration with the client on tasks or goals of therapy and strengthen the emotional bond (Eubanks and Muran 2022). Two meta‐analyses have been conducted exploring the association between rupture repair processes and treatment outcomes (Eubanks et al. 2018; Safran et al. 2011). Both meta‐analyses found a moderate positive association between rupture repair and treatment outcomes, with pooled effect sizes of *r* = 0.24 (Safran et al. 2011) and *r* = 0.29 (Eubanks et al. 2018). Although many studies on alliance ruptures have been published in recent years, the field still lacks a meta‐analytic investigation of the direct relationship between alliance ruptures and treatment outcomes. Understanding the size and consistency of the association between alliance ruptures and poorer treatment outcomes is essential to contextualize the benefits of rupture repair.

Studies investigating ruptures rely on a variety of measurement instruments, which can be distinguished conceptually and operationally as direct or indirect measures (Eubanks et al. 2018). Direct rupture measures are specifically designed to assess rupture processes—such as withdrawal or confrontation—either through self‐report or observer‐based coding. Self‐report measures, such as the Post‐Session Questionnaire (PSQ; Muran et al. 1992), ask clients or therapists to explicitly report on the occurrence or intensity of ruptures (e.g., “Did you experience any tension, misunderstanding, conflict, or disagreement in your relationship with your therapist/patient during the session? If yes, please rate how tense or upset you felt about this during the session.”). Observer‐based methods, such as the Rupture Resolution Rating System (3RS; Eubanks et al. 2018; Eubanks and Muran 2022), involve independent coders assessing videotaped therapy sessions for withdrawal and confrontation ruptures (e.g., Babl et al. 2022). Direct measures often allow for more fine‐grained, episode‐level assessment, capturing subtle or transient ruptures that may be repaired within the same session (Babl et al. 2024; Coutinho et al. 2014).

In contrast, indirect rupture measures infer rupture processes from fluctuations or declines in alliance ratings rather than measuring ruptures explicitly. For example, the Working Alliance Inventory (WAI; Tracey and Kokotovic 1989) has frequently been used as an indirect rupture measure, where drops in session‐level alliance scores are interpreted as potential rupture events (Eubanks‐Carter et al. 2012). Comparisons between WAI‐based indicators and the 3RS suggest that alliance fluctuations capture some rupture events but may miss more subtle or transient disruptions, such as withdrawal ruptures or ruptures that are repaired within a session (Babl et al. 2024; Coutinho et al. 2014). Importantly, these findings primarily concern indirect, alliance‐fluctuation measures and do not necessarily generalize to self‐report measures designed to explicitly assess rupture experiences, such as the PSQ.

This conceptual and operational distinction highlights that while some items on alliance measures may overlap with rupture experiences, direct measures are specifically tailored to identify rupture processes at the session or episode level, whereas indirect measures infer ruptures from broader alliance changes.

Based on the gaps in previous research on alliance ruptures, the current meta‐analysis aims to establish a link between ruptures (assessed with direct measures) and outcome. It was hypothesized that alliance ruptures would be negatively associated with psychotherapy outcomes.

## 2 Methods

The current meta‐analysis followed the Preferred Reporting Items for Systematic Reviews and Meta‐Analyses (PRISMA; Page et al. 2021) guidelines.

### 2.1 Search Strategy

Through the research platform EBSCOhost, the database PsycINFO was comprehensively screened. The search terms were applied in a multi‐field format in the following manner: *alliance* (field 1) AND *rupture* (field 2) AND *psychotherapy* OR *therapy* OR *counseling* OR *intervention* OR *treatment* (field 3). All three fields must be mentioned in the abstract and search options were specified to *All Journals*, *Journal Article* and *English language*. This primary search resulted in a list of 232 publications on January 16, 2024.

### 2.2 Eligibility Criteria

Inclusion and exclusion criteria of the meta‐analysis were as follows: (a) be an empirical study (not a review or theoretical paper), (b) not be a case study or task analysis, (c) involve a quantitative and direct measure of alliance ruptures, (d) involve a quantitative outcome measure, (e) implement a psychotherapeutic intervention (in contrast to primary medical care (or other types of intervention), (f) include a population with a sample mean age of at least 18 years, (g) not be duplicated data, and (h) report an effect size of the association between ruptures and psychotherapy outcome.

### 2.3 Selection Process

Screening was conducted in two sequential stages: (1) title‐and‐abstract screening and (2) full‐text screening. Both stages were carried out independently by three master's‐level psychology students. Following each stage, discrepancies regarding study eligibility were resolved through discussion with the supervisor until consensus was reached.

**Figure 1:**
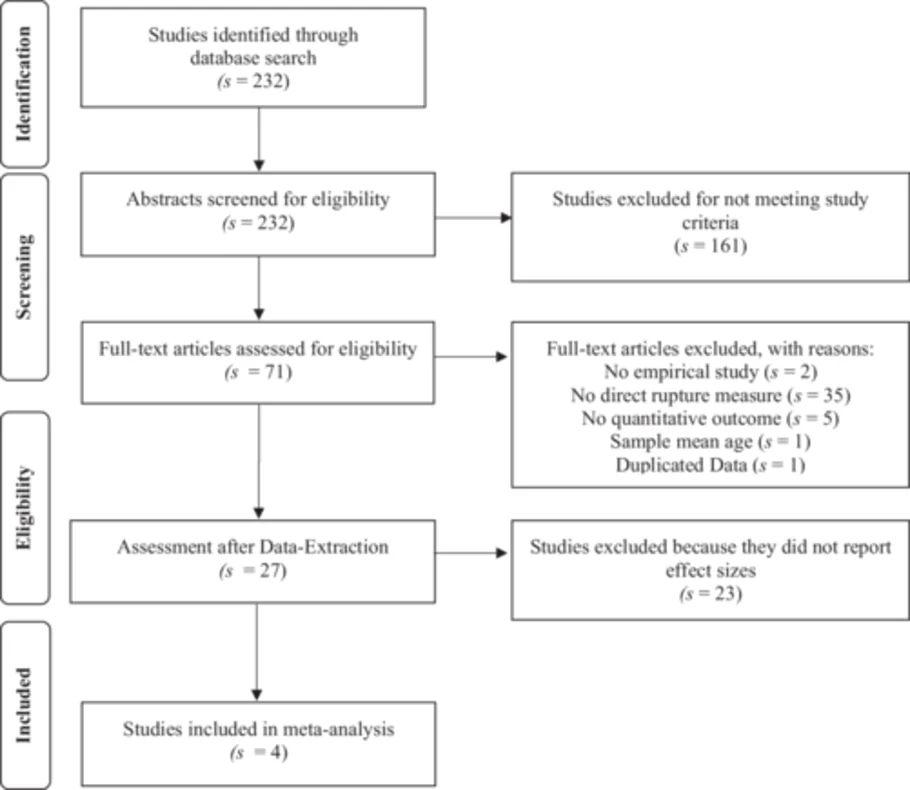
Flow chart of the studies included in the meta‐analysis.

The initial search of the PsycINFO database using the predefined search strategy yielded 232 publications. All records underwent title‐and‐abstract screening, followed by full‐text screening for potentially eligible studies by three independent coders. Inclusion and exclusion decisions were based on the predefined eligibility criteria. Interrater reliability was assessed using Fleiss' κ, a chance‐corrected measure of agreement for multiple raters. For the title/abstract screening stage, agreement was moderate/substantial (Fleiss' κ = 0.49) and 161 articles were excluded. The remaining 71 articles went for the full‐text screening stage, agreement was moderate (Fleiss' κ = 0.34). An additional 44 studies were excluded. Of the 27 remaining studies, four reported effect sizes for the association between ruptures and psychotherapy outcomes and were thus included in the meta‐analysis. For the flow‐chart of the studies included in this meta‐analysis, see Figure 1.

Finally, in an effort to find relevant studies that might have been uploaded on other databases or were inadvertently omitted due to our search terms, we manually examined the reference lists of the 27 eligible publications. This secondary screening did not yield additional studies. General descriptive data of the studies meeting the inclusion criteria for this meta‐analysis are provided in Table 1.

**Table 1:** Main characteristics of the studies included in the meta‐analysis.

Authors (year)	Peer‐Reviewed	Treatment type/treatment conditions	Average treatment length (in sessions)	*n* clients	Mean client age (SD)	% Female clients	*n* therapists	Mean therapist age (SD)	Client main diagnosis
Atzil‐Slonim et al. (2021)	Yes	Psychodynamic psychotherapy	15	58	39.06 (13.67)	58.9%	52	N/A (N/A)	Mostly anxiety and affective disorders
Watson et al. (2017)	Yes	Sex offender treatment program	36	71	45.06 (15.07)	0.0%	17	N/A (N/A)	Sex offense
Gersh et al. (2017)	Yes	Two conditions: Cognitive analytic therapy or Befriending treatment	16	44	18.2 (2.81)	81.8%	14	N/A (N/A)	Borderline personality disorder
Muran et al. (2009)	Yes	Three conditions: Short‐term psychodynamic therapy or BRT or CBT	30	128	41.33 (10.52)	53.1%	70	38.14 (1.89)	Personality disorders

Abbreviations: BRT = brief relational therapy, CBT = cognitive behavioral therapy.

### 2.4 Data Extraction

Data from eligible publications was extracted independently by three master‐level psychology students using a data extraction form specifically designed for the purpose of this study. The extracted information of the three coders was subsequently compared, and any disagreements were discussed collaboratively with the supervisor until consensus was reached. No formal interrater reliability statistic was calculated.

The data extraction form comprised the following information: authors, year of publication, title of the study, sample characteristics including sample size, mean age, percentage of female clients, primary diagnosis, and prevalence of personality disorder diagnoses; therapist characteristics including mean age and percentage of female therapists; therapy length; number of sessions per week; method used to measure alliance ruptures; and timing of the rupture assessment. Additionally, information regarding the method used to assess treatment outcome, timing of outcome assessment, comparison groups, and reported effect sizes was extracted.

Some aspects of the extraction process required researcher judgment, particularly the classification of how alliance ruptures were operationalized across studies. Specifically, effect sizes reflected rupture intensity, rupture frequency, or the presence versus absence of a rupture. Rupture intensity was the most common operationalization. Judgment was also required when interpreting study descriptions to determine the relevant timing and measurement procedures for ruptures and outcomes.

### 2.5 Statistical Analysis

The analyses were conducted within the free environment *R* (R Core Team 2021) with the package *metafor* (Viechtbauer and Cheung 2010). We used a multilevel meta‐analytic model with a three‐level structure with a restricted maximum likelihood method to estimate the aggregated effect size of ruptures on outcome. In this model, sampling variance (Level 1) was nested within effect sizes (*k* = 18; Level 2), nested within studies (*s* = 4; Level 3). Of the four included studies, three reported effect sizes as Pearson's *r*. The Adjusted *R* ^2^ originally reported in the other study was converted into Pearson's *r* by use of an online converter (Lin n.d.). A multilevel forest plot was used to illustrate the distribution of the effect sizes.

Most studies reported multiple effect sizes, for instance when measuring rupture frequency from both patients' and therapists' perspectives or when measuring several outcome variables. Thus, to assess the overall heterogeneity across the included effect sizes, a *Q* analysis was implemented (Higgins and Thompson 2002). We have harmonized effect directions across studies. We decided to code effect directions so that a negative correlation coefficient (*r*) consistently represents a negative association between alliance ruptures and psychotherapy outcomes; that is, higher levels of alliance ruptures are associated with poorer treatment outcomes. Where necessary, correlations were reversed to ensure that the direction of effects was comparable across studies, regardless of the original scoring direction of the outcome measures (Table 2). The correlation coefficients were transformed into Fisher's *z* and the analysis was performed based on those z scores (Borenstein et al. 2010). Then, the *z* was transformed back into correlation coefficients in order to allow for easier interpretation.

**Table 2:** Characteristics of the effect sizes reported in the included studies of the meta‐analysis.

Authors (year)	Rupture measure	Timing rupture measure	Outcome (measure)	Timing outcome measure	Outcome rater	Reported ES	*r* (negative *r* values represent a negative rupture–outcome association)
Atzil‐Slonim et al. (2021)	PSQ‐T (presence of rupture)	After each session	Symptomatology (HSCL)	Before each session	Patient	*r* = 0.32	−0.32
Watson et al. (2017)	PSQ‐P (presence of rupture)	¾ through treatment	Treatment gain (TRRG‐SV)	Post‐treatment	Other	Adjusted *R* ^2^ = −0.00	−0.00
Gersh et al. (2017)	3RS (number of ruptures) (rupture intensity)	Sessions 3, 9, 15	Interpersonal problems (SAS‐SR)	Pre–post change scores	Patient	Early session (3): *r* = 0.32	−0.32
						Mid session (9): *r* = 0.28	−0.28
						Late session (15): *r* = −0.30	0.30
						Early session (3): *r* = 0.25	−0.25
						Mid session (9): *r* = 0.16	−0.16
						Late session (15): *r* = −0.24	0.24
	3RS (number of ruptures) (rupture intensity)		Symptomatology (BPDSI‐IV)	Pre–post change scores	Other	Early session (3): *r* = 0.18	−0.18
						Mid session (9): *r* = 0.42	−0.42
						Late session (15): *r* = 0.43	−0.43
						Early session (3): *r* = 0.17	−0.17
						Mid session (9): *r* = 0.36	−0.36
						Late session (15): *r* = 0.40	−0.40
Muran et al. (2009)	PSQ‐P (rupture intensity)	After sessions 1–6 (average)	Symptomatology (SCL‐90‐R & GAS)	Post‐treatment	Patient	*r* = −0.15	−0.15
	PSQ‐T (rupture intensity)		Symptomatology (SCL‐90‐R & GAS)	Post‐treatment	Therapist	*r* = −0.08	−0.08
	PSQ‐P (rupture intensity)		Interpersonal problems (IIP & WISPI)	Post‐treatment	Patient	*r* = −0.35	−0.35
	PSQ‐T (rupture intensity)		Interpersonal problems (IIP & WISPI)	Post‐treatment	Therapist	*r* = −0.32	−0.32

Abbreviations: BPDSI‐IV = borderline personality disorder severity index‐IV, ECR = experience in close relationships scale, GAS = global assessment scale, HSCL = hopkins symptom checklist, IIP = inventory of interpersonal problems, PSQ‐P = post‐session questionnaire patient version, PSQ‐T = post‐session questionnaire therapist version, SAS‐SR = social adjustment scale—self‐report, SCL‐90‐R = symptom checklist‐90‐revised, TRRG‐SV = treatment readiness responsivity gain scale—short version, WISPI = wisconsin personality inventory.

The magnitudes of the correlations were classified as follows: small effect size (*r* = 0.10), medium effect size (*r* = 0.30), and large effect size (*r* = 0.50) (Cohen 1992). In an effort to check for publication bias, a funnel plot which depicts the standard error (vertical axis) and effect sizes for each study (horizontal axis; Fernández‐Castilla et al. 2020) was created and analyzed. A clear asymmetry towards one side of the mean represents outliers and would be indicative of publication bias. No such pattern could be detected in our sample.

## 3 Results

### 3.1 Study and Effect Size Characteristics

The four studies that met the inclusion criteria for the meta‐analysis provided data from *N* = 301 clients undergoing treatment, including a total of 18 correlational effect sizes of the association between alliance ruptures and psychotherapy outcome. The studies included treatments from different theoretical frameworks, such as psychodynamic psychotherapy, cognitive‐analytic therapy, befriending treatment, brief relational therapy, cognitive behavioral therapy and a sex offender treatment program. Main diagnoses ranged from anxiety and affective disorders to paraphilia and personality disorders.

Studies used either the 3RS or PSQ as direct measures of rupture (most of the time repeatedly in the early stages of treatment), alongside a range of outcome measures (most of the time post‐treatment scores). Assessment timing varied across studies, as reported in Table 2. Treatment length ranged from 15 to 36 weekly sessions (26 on average), as presented in Table 1. All studies were peer‐reviewed. Furthermore, characteristics of the effect sizes included in this meta‐analysis are provided in Table 2. The study quality of the included studies ranged from 67% to 83% (the criteria and quality rating per study can be found in the Supporting Information S1).

### 3.2 Meta‐Analytic Models

**Figure 2:**
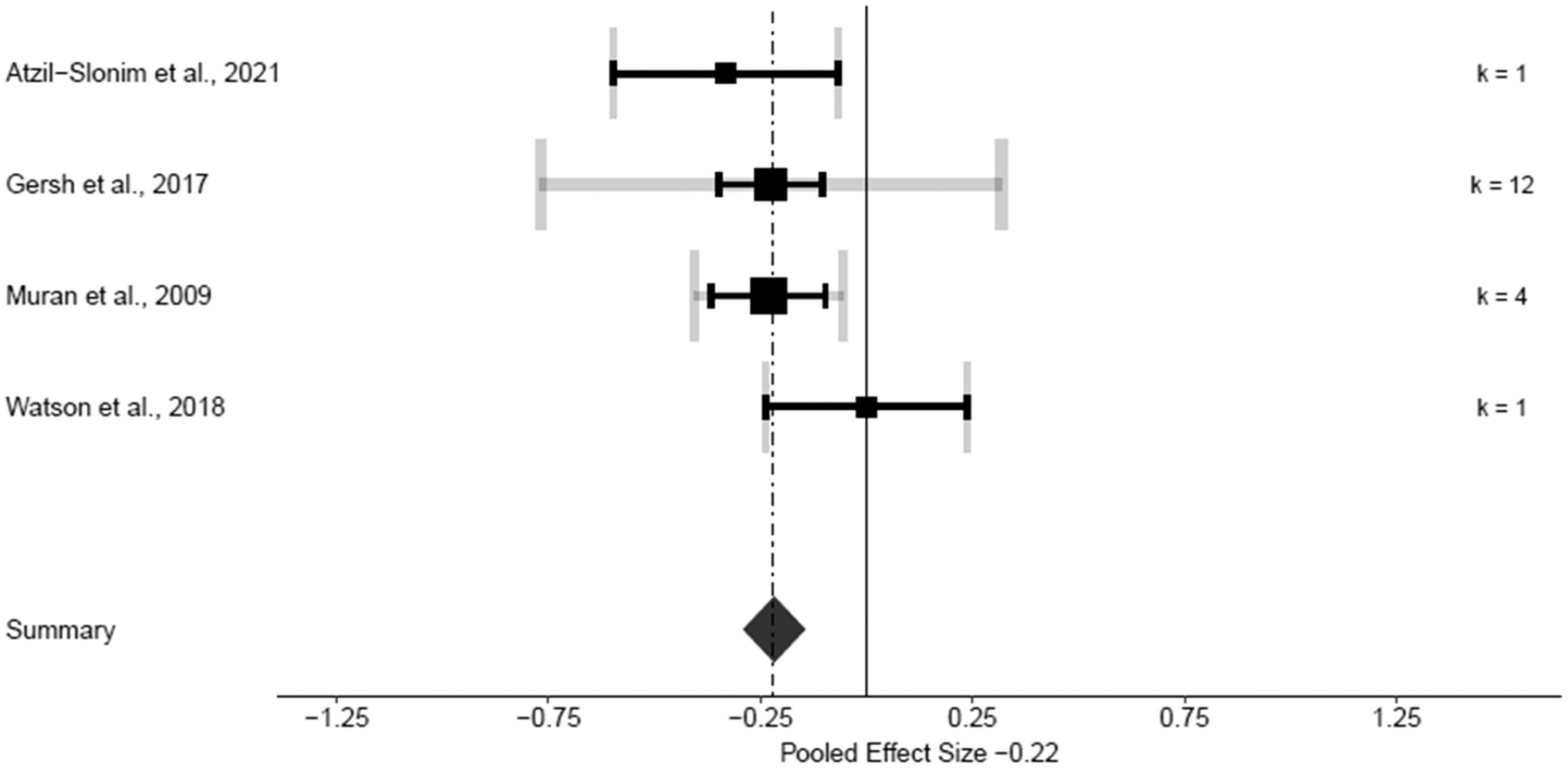
Forest plot of the pooled correlational (*r*) effect sizes representing the association between ruptures and outcomes. The gray confidence interval represents the sampling variance (Level 1). The black confidence interval represents the within‐study variance (Level 2).

The results of the three‐level model showed a significant aggregated effect size of *z* = −0.22, *SE* = 0.04, 95 CI [−0.303, −0.133]; *t*(17) = −5.40, *p* < 0.001, *r* = −0.21, demonstrating a significant negative association between alliance ruptures and treatment outcomes. In Figure 2, a forest plot is presented displaying the effect sizes with their sampling variance, within‐study variability and between‐study variability.

The *Q* test indicated that heterogeneity across effect sizes was not statistically significant, *Q*(17) = 19.58, *p* = 0.30. Variance decomposition showed that 20.02% of the total variance was attributable to within‐study variability across reported outcomes (Level 2), whereas < 1% was attributable to between‐study variability (Level 3). These estimates descriptively suggest greater variability within studies than between studies; however, this interpretation should be treated with caution. Given the small number of included studies (*s* = 4), the between‐study variance estimate is likely unstable, and the analysis has limited power to detect between‐study heterogeneity.

**Figure 3:**
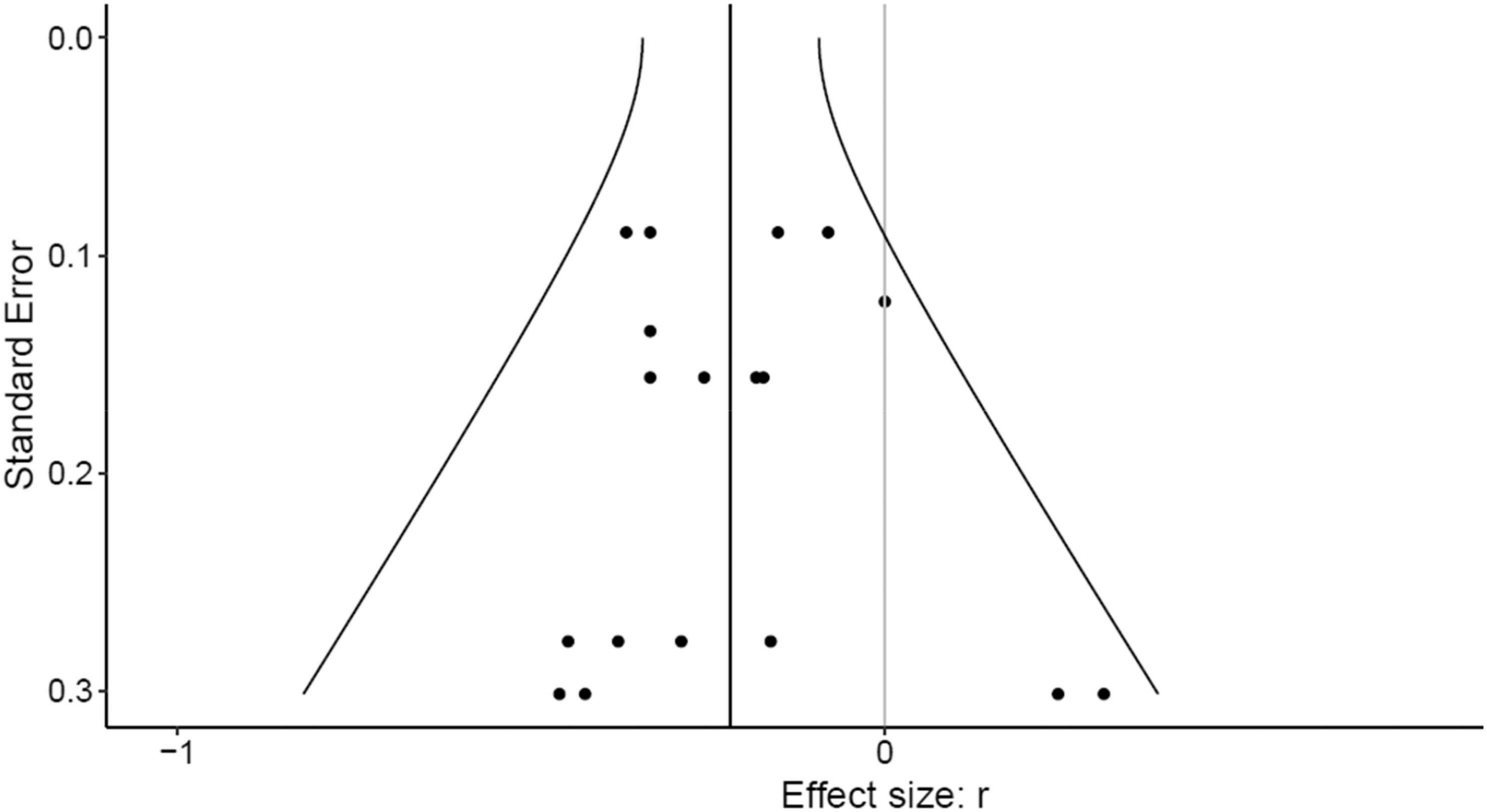
Funnel plot of aggregated effect sizes (*k* = 18) within studies (*s* = 4).

The funnel plot displayed in Figure 3 did not show evidence of substantial publication bias. The absence of outliers and the plots tending towards the same direction (Figure 2) indicate a small but consistent effect size estimation.

## 4 Discussion

This is the first meta‐analysis analyzing the association between alliance ruptures and outcome in psychotherapy. We found a small but consistent negative association between alliance ruptures and client outcome, drawing exclusively from studies that employed direct measures of ruptures.

In line with our hypothesis and previous findings (e.g., Rubel et al. 2018), the results suggest that ruptures during the therapeutic process are associated with worse therapy outcomes. Importantly, previous research has suggested that the consequences of alliance ruptures may depend on how they are addressed within therapy (Eubanks et al. 2018; Safran et al. 2011). However, the present meta‐analysis did not examine rupture repair and therefore cannot determine whether repair moderates the association between ruptures and outcome. Future research should investigate whether repaired and unrepaired ruptures differ in their associations with treatment outcome and under which conditions rupture repair may contribute to therapeutic change.

The present findings should be interpreted in light of substantial heterogeneity across study characteristics. Variations in clinical populations (e.g., diagnosis, severity) or therapeutic modality (e.g., CBT, psychodynamic) could influence the magnitude of rupture–outcome associations. Certain populations or treatment approaches may be more sensitive to the impact of ruptures, and ruptures that are assessed more frequently or at the session level may be more strongly related to outcomes.

An additional source of heterogeneity concerns the operationalization of alliance ruptures. Although all included studies used direct rupture measures, the synthesized effect sizes did not uniformly represent the same rupture characteristic. Most effect sizes reflected rupture intensity, whereas others reflected rupture frequency or the mere occurrence of ruptures. Consequently, the pooled estimate should be interpreted as reflecting the overall burden of alliance ruptures rather than a specific rupture dimension. Future research should examine whether rupture intensity, frequency, and occurrence differ in their associations with psychotherapy outcome.

The interpretability of the results is further complicated by the diversity of outcome measures, assessment timing (e.g., before each session, pre–post change scores), and frequency (e.g., repeated measures vs. a single timepoint) used across the included studies, which precluded meaningful comparisons.

While our pooled estimate suggests a small‐to‐moderate negative association between ruptures and outcome, this effect likely reflects an average across diverse contexts, and variability across studies may not be fully captured due to limited sample size. Although we restricted inclusion to studies using direct rupture measures, future meta‐analyses with a larger number of studies are needed to enable a more statistically robust synthesis and to explore sources of heterogeneity through moderator (e.g., outcome type, outcome rater), subgroup or sensitivity analyses.

Future research should include the 23 studies that fulfilled all other inclusion criteria but did not report effect sizes for the association between ruptures and psychotherapy outcome. Incorporating these studies, for example, through an individual patient data (IPD) meta‐analysis, would increase statistical power and provide a more comprehensive understanding of the rupture–outcome relationship. An IPD meta‐analysis would further allow to disentangle the effects of repaired and unrepaired ruptures on psychotherapy outcome, by indeed including rupture repair as a moderator of the rupture effect on outcome which unfortunately was not possible in this traditional meta‐analysis due to the presentation of the results in the original studies.

More generally, the temporal ordering of rupture and outcome processes remains unclear across much of the included literature. Because most studies assessed associations between alliance ruptures and treatment outcomes without explicitly examining their temporal sequencing, the observed relationships should not be interpreted as evidence that ruptures necessarily cause poorer outcomes. Alternative explanations remain possible, including the possibility that clients who are progressing less favorably may be more likely to experience difficulties within the therapeutic relationship and subsequent rupture experiences. As a result, the direction of influence between ruptures and treatment outcomes cannot be established from the available evidence. Future research should employ longitudinal designs with repeated assessments of both rupture processes and clinical outcomes to clarify their temporal and potentially reciprocal relationships.

In conclusion, this meta‐analysis adds to the existing literature on alliance ruptures by providing evidence for a negative association between ruptures and therapy outcome using direct rupture measures. Reporting standards with regard to rupture data in this young but promising field need to be established in order to lay ground for comparisons between studies.

## Ethics Statement

This study is a meta‐analysis based exclusively on data reported in previously published studies. No new data involving human or animal participants were collected; therefore, ethical approval was not required.

## Supporting information


Supporting File

## Data Availability

Data sharing not applicable to this article as no datasets were generated or analyzed during the current study.
