# Supplementary material for: Alliance Ruptures and Psychotherapy Outcomes: A Multilevel Meta‐Analysis of Their Association
Source: J Clin Psychol. 2026 Jul 17;82(10):1683–91. doi: 10.1002/jclp.70192 (PMC13544451; doi:10.1002/jclp.70192)
Supplement: Supplementary file 1 [file JCLP-82-1683-s001.odt]

Supplementary Material  A
Quality Assessment of Included Studies

Table S1. Quality assessment of included studies
Study
Designs
Sample size
Dropout
Assessment
Reliability Measurement

Quality

Q1
Q2
Q3
Q4
Q5.a
Q5.b

Atzil-Slonim et al. (2021)
1
0
1
0
 1*
1
67
Gersh et al. (2017)
1
0
1
1
1
 1*
83
Muran et al. (2009)
1
1
1
0
 1*
 1*
83
Watson et al. (2018)
1
1
1
0
 1*
 1*
83


Note. * = not assessed in this study sample but assessment of previous studies demonstrated adequate, sound, or good reliability


Quality Assessment Criteria

Question 1 – Design
Does the study have a prospective or longitudinal design?
No, variables of interest were measured at the same time point
(1) Yes

Question 2 – Power/ Sample size
In order to find a correlation (r) of .30 with a power of 80 and an alpha level of .05, one would need a sample of 64 participants. Is the sample large enough to detect the expected association?
(0) No, sample size is smaller than 64
(1) Yes, the sample consists of 64 participants or more

Question 3 – dropout/attrition
Was there less than 20 % of dropout?
(0) No, there was more than 20% attrition, or they have not mentioned how much attrition they specifically had
(1) Yes, there was less than 20% of attrition

Question 4 – Assessment method of Rupture
Was an “objective” measure used to assess “alliance ruptures”:
(0) No, ruptures were assessed via self-report measures
 (1) Yes, ruptures were assessed via observed scores from independent coders/ experimenters

Question 5 - Reliability of Variables of interest

a) Was the measurement of  the variable “ alliance ruptures” reliable?
(if the reliability is not assessed in the study sample, check previous studies that assessed psychometric properties of instrument in question)

(0) No, the ICC (inter-coder reliability) or alpha was <.60 or in the text ‘low reliability’ was mentioned or they do not mention reliability at all (also not previously assessed)
Yes, the reliability for the predictor measure is higher than >.60


b) Was the measurement of the study outcome variable reliable?
(0) No, the ICC (inter-coder reliability) was <.60 or in the text there was something like ‘low reliability’ or they do not mention reliability at all (also not previously assessed)
(1) Yes, the reliability for the outcome measure is higher than >.60

Calculation of Quality score:
Add all points together and weigh score (divide by number of questions answered)
